# Slovenian Validation of the Children’s Perceived Use of Self-Regulated Learning Inventory

**DOI:** 10.3389/fpsyg.2021.730386

**Published:** 2022-01-14

**Authors:** Luka Komidar, Anja Podlesek, Tina Pirc, Sonja Pečjak, Katja Depolli Steiner, Melita Puklek Levpušček, Alenka Gril, Bojana Boh Podgornik, Aleš Hladnik, Alenka Kavčič, Ciril Bohak, Žiga Lesar, Matija Marolt, Matevž Pesek, Cirila Peklaj

**Affiliations:** ^1^Department of Psychology, Faculty of Arts, University of Ljubljana, Ljubljana, Slovenia; ^2^Educational Research Institute, Ljubljana, Slovenia; ^3^Department of Textiles, Graphic Arts and Design, Faculty of Natural Sciences and Engineering, University of Ljubljana, Ljubljana, Slovenia; ^4^Laboratory of Computer Graphics and Multimedia, Faculty of Computer and Information Science, University of Ljubljana, Ljubljana, Slovenia

**Keywords:** self-regulated learning, primary education, test adaptation, validity, measurement invariance

## Abstract

The importance of self-regulated learning (SRL) has increased during the COVID-19 pandemic and measures for assessing students’ self-regulation skills and knowledge are greatly needed. We present the results of the first thorough adaptation of the Children’s Perceived use of Self-Regulated Learning Inventory (CP-SRLI). The inventory, consisting of 15 scales measuring nine components of SRL, was administered to a sample of 541 Slovenian ninth graders. Confirmatory factor analyses supported internal structure validity of most components, but two components required some structural modifications. Internal consistency coefficients were acceptable for the majority of scale scores and were highly comparable to the original ones. While metric invariance across gender was confirmed, the scalar invariance of some scales needs further examination. Meaningful correlations with relevant externally assessed and self-reported self-regulation and school performance variables indicated good criterion validity of the inventory. The Slovenian version of the CP-SRLI thus proved to be a sufficiently valid and reliable instrument for assessing pupils’ learning self-regulation.

## Introduction

The COVID-19 pandemic profoundly changed our lives, which was particularly evident in the area of education. Many countries had to change their entire education system to distance learning for several months during the first and second waves of the pandemic. In this situation, students faced enormous challenges regarding their learning. Classes moved to the online environment, the amount of written communication increased tremendously, they had to use more digital sources, the opportunities to get immediate help from their teachers decreased, they had to plan their own school day, and avoid various distractions in their home environment. The novelty and stressfulness of this situation emphasized the importance of self-regulated learning (SRL). To help students become successful self-regulated learners, we need valid and reliable tools to assess their strengths and weaknesses in SRL, especially for students in elementary and secondary schools who are still developing such skills.

### The Concept of Self-Regulated Learning

There are several major theories of SRL that emphasize the multicomponent nature of SRL. For example, Pintrich (2000, 2004) defined SRL as an active process in which individuals set goals, monitor their learning, and regulate it according to goals and contextual demands. Using feedback loops, students check if their learning methods and strategies lead to their goals, and modify them accordingly (Carver and Scheier, 1981, 2011). Models based on the socio-cognitive perspective include (meta) cognitive and motivational processes of SRL (Garcia and Pintrich, 1994; Boekaerts, 1997; Zimmerman, 2000; Zimmerman and Moylan, 2009) and subsequently added behavioral and contextual factors (Pintrich, 2000, 2004). Cognitive components include content knowledge, metacognitive knowledge about self and tasks, and cognitive and metacognitive strategies. Motivational components refer to students’ goal orientation, interest, task value, self-efficacy, and motivational regulation strategies. In her metacognitive and affective model of self-regulation (MASRL model), Efklides (2011) extended the previously mentioned models by highlighting two levels of SRL, namely the personal level, which includes more stable personal characteristics (ability, motivation, affect, self-concept, control beliefs, metacognitive knowledge, and metacognitive experiences), and the Task × Person level, which includes task processing and subjective experiences that interact and inform each other in SRL.

In the self-determination theory, Ryan and Deci (2000) highlighted three sources of individual motivation to act, i.e., the need for autonomy, a sense of competence, and relatedness. They presented different regulatory styles of motivation and treated it as a continuous variable ranging from external motivation to introjected, identified, and integrated motivation. These components may be reflected in different phases of self-regulation before, during, and after learning, namely in forethought (planning and activation), monitoring, control, and reflection (Pintrich, 2004).

### Children’s Perceived Use of Self-Regulated Learning Inventory

To measure SRL skills and knowledge, several instruments were developed. For example, the Learning and Study Strategies Inventory (LASSI; Weinstein et al., 1987) is a tool for assessing college students’ awareness about and the use of learning strategies related to skill, will, and self-regulation components of strategic learning. The Motivated Strategies for Learning Questionnaire (MSLQ; Pintrich et al., 1993) was developed to measure the types of learning strategies and academic motivation in college students. The Children’s Perceived use of Self-Regulated Learning Inventory (CP-SRLI; Vandevelde et al., 2013) measures nine components of SRL in primary school pupils.

The CP-SRLI includes 15 scales reflecting nine components: planning, motivation (extrinsic, introjected, identified, intrinsic), self-efficacy (regulation, motivation), monitoring, learning strategies (deep-level and surface strategies), motivational strategies, persistence, product self-evaluation, and process self-evaluation. The original validation study by Vandevelde et al. (2013) examined the internal structure validity on a sample of 409 fifth and 314 sixth graders from 45 classes from 17 inner-city Flemish primary schools, with a mean age of all participants being 10.9 years. Because item responses were not normally distributed, they used the robust maximum likelihood estimator (MLR; Yuan-Bentler correction). The fit of the models for the nine CP-SRLI components was generally satisfactory, and ranged from excellent (e.g., CFI > 0.95, RMSEA < 0.05) to acceptable (e.g., CFI > 0.90, RMSEA < 0.08). To achieve an acceptable fit, they had to estimate correlations between residuals of some item-pairs from Self-Efficacy, Monitoring, and Motivational Strategies (for details see Vandevelde et al., 2013). Most scale scores had acceptable reliability, except for Planning (Bentler’s ρ = 0.54), Self-Efficacy Motivation (ρ = 0.62), and Motivational Strategies (ρ = 0.65). The results of their multiple-group factor analyses supported metric and scalar invariance of the scales across gender. Due to poor internal consistency of some CP-SRLI components, the questionnaire is most useful for research purposes. However, it can still be used by school counselors to identify the students’ (meta) knowledge, strategies, and skills that should be systematically developed or could be further improved.

We decided to conduct a Slovenian adaptation of the CP-SRLI for several reasons. First, the CP-SRLI is the first holistic multicomponent self-report measure of SRL that is well grounded in the socio-cognitive models of SRL, e.g., the models developed by Boekaerts (1997), Pintrich (2004), and Zimmerman and Moylan (2009). Additionally, it includes a disaggregated construct of motivation that includes four types of motivation: extrinsic, introjected, identified, and intrinsic (Deci and Ryan, 2002). Second, most of the existing SRL instruments, e.g., LASSI (Weinstein et al., 1987) and MSLQ (Pintrich et al., 1993), are more appropriate for high school and college students than primary school students. Due to limited research on primary school children’s SLR, Vandevelde et al. (2013) validated the CP-SRLI on fifth and sixth graders (aged between 10 and 12 years), who are approaching the transition from primary to secondary school in Flemish education. In our adaptation of the CP-SRLI, we decided to focus on older primary school children (i.e., ninth graders in Slovenian primary schools who are 14–15 years old) who are approaching the transition to high school in the Slovenian educational system (in Slovenia, primary school has nine grades). Well-developed SLR skills of students can ease their transition to secondary schools, where they encounter increasingly demanding subjects and have to rely more on independent learning from textbooks and other sources (books, papers, websites, etc.). Third, the CP-SRLI mainly focuses on schoolwork at home, where the impact of students’ self-regulatory skills is crucial. We also chose this age range because neurological studies showed that most cognitive control, which is a key component of self-regulation, is developed before the age of 13 (Fjell et al., 2012), and because of the results of developmental studies showing intensive development of metacognition in 12–14-year-olds (Van der Stel and Veenman, 2010).

Numerous studies have already used the CP-SRLI for investigating SRL, but we could not find any validation studies of the instrument. To our knowledge, the present study represents the first rigorous validation of the CP-SRLI in a different linguistic environment. Our goals were to translate the CP-SRLI into Slovenian and to evaluate the internal structure validity, reliability, measurement invariance across gender, and criterion validity of the Slovenian version. We decided to assess the latter by examining the relationships between the CP-SRLI scores and measures of metacognitive knowledge, academic achievement, and time management.

### The Relationship of Self-Regulated Learning With Academic Achievement, Time Management, and Metacognitive Knowledge

Research on SRL has shown that it is one of the most important predictors of different academic achievement outcomes, such as GPA, test scores, final grades etc. (Hattie, 2009; Dent and Koenka, 2016). Correlations between different components of SRL and academic achievement vary considerably (Veenman et al., 2014; Dent and Koenka, 2016). Regarding individual cognitive strategies, the highest positive correlations with academic achievement were found for elaborative and organizational strategies (Dent and Koenka, 2016), and for planning and monitoring (Greene and Azevedo, 2009; Roebers et al., 2014; Dent and Koenka, 2016). For the motivational components of SRL, self-efficacy (Bembenutty and Zimmerman, 2003; Komarraju and Nadler, 2013; Jackson, 2018), interest (Bembenutty and Zimmerman, 2003), task value (Komarraju and Nadler, 2013), and effort regulation (Jackson, 2018) were found to have the highest positive correlations with academic achievement. In addition, Heirweg et al. (2019) found a positive relation between well-developed self-regulatory skills and test performance in a sample of Dutch 10–11 year-old students.

At the behavioral level, students’ SRL skills are reflected in their time management of daily activities and of schoolwork in general, time management when preparing for tests, regularity of schoolwork, and in their academic/learning performance. Time is one of the essential resources in learning, so a time management strategy can be seen as a source management strategy (McInerney, 2013). Students have to balance between academic and extracurricular activities. This can be challenging for many students, especially for those who take part in numerous or time-consuming extracurricular activities. For some students, organizing their schedule can lead to increased stress, emotional problems, and lower achievements (Indreica et al., 2011; Cyril, 2015; Núñez et al., 2015). In contrast, well-developed time management skills enable students to plan their own learning and other activities, choose goals, and prioritize different tasks (e.g., when preparing for tests). Students with such skills are well prepared, organized, and focused in managing their daily lives and completing academic tasks in a timely manner (Cyril, 2015). Time management is also closely related to regularity of schoolwork (doing homework and learning in general). Well-developed time management skills assist students to complete assignments more regularly. Research shows that regularity of homework completion leads to better learning achievements (Trautwein, 2007; Núñez et al., 2015; Tenko, 2019).

Academic achievement is also associated with metacognitive knowledge. To use self-regulatory skills, students need to have knowledge about themselves (how they function as students), the learning tasks and goals, and the use of cognitive and metacognitive strategies (Flavell, 1979; Efklides, 2011). In a sample of primary school students, Özsoy et al. (2009) found a significant relationship between metacognitive awareness and academic achievement for successful students, but not for medium or low achieving students. Sawhney and Bansal (2015) found similar results for undergraduate students, i.e., students with higher metacognitive awareness had higher academic achievement. In addition, the results from a study by Bogdanović et al. (2017) indicated that 15-year-old students with better metacognitive knowledge had higher achievement in physics. Similar was true for 11-year-old students in mathematics (Özsoy, 2011).

Because research has shown important relationships between metacognitive knowledge and achievement, and between behavioral-level self-regulation and academic achievement, we used metacognitive knowledge, time management of daily activities, schoolwork, studying for tests, regularity of schoolwork, and average school grade in the previous year (as a measure of academic achievement) as criterion variables for validating the CP-SRLI. In addition, we included two other criterion variables related to time management for extracurricular activities (in-school and out-of-school) that were also found to be related to students’ daily self-regulation (e.g., Carroll and Purdie, 2007).

## Materials and Methods

### Participants

The sample obtained by convenience sampling consisted of 541 ninth graders (aged 14–15 years) from 18 Slovene primary schools that agreed to participate in our study. The schools were of different sizes and were dispersed across all twelve Slovenian regions. Five schools were from rural and 13 from urban areas. In Slovenia, almost all schools are public (99.2%, Taštanoska, 2019), and are attended by students from all socioeconomic classes (there is no segregation by socioeconomic status [SES]). Since the entire school classes were included in our study, it is highly likely that the SES of the students in our sample was representative of the students’ SES in the reference population. Girls (*n* = 268) and boys (*n* = 271) were equally represented in the sample.

### Instruments

#### Children’s Perceived Use of Self-Regulated Learning Inventory

The CP-SRLI (Vandevelde et al., 2013) is a 75-item self-report questionnaire comprised of nine components (Table 1) that pertain to cognitive (task orientation, learning strategies), metacognitive (planning, persistence, monitoring, self-evaluation), and motivational (motivation, motivational strategies, self-efficacy) aspects of SRL. Participants provide responses on a 5-point scale ranging from “1 = completely untrue for me” to “5 = completely true for me.” The majority of (sub) component scores exhibited satisfactory reliability (Bentler’s ρ ranging from 0.54 to 0.85) in the original validation study (Vandevelde et al., 2013). The sub (component) scores can be calculated as a sum or an average of the corresponding item responses.

**TABLE 1:** CP-SRLI components’ basic characteristics and shape of item distributions.

CP-SRLI component	No. of scales	No. of items	No. of missing values (%)	Skewness *M* (range)	Kurtosis *M* (range)
Task orientation	1	6	15 (0.46%)	–0.24 (–0.78, 0.22)	–0.55 (–1.08, 0.14)
Planning	1	4	11 (0.51%)	–0.59 (–0.95, 0.07)	–0.34 (–1.12, 0.12)
Motivation	4	14	20 (0.26%)	–0.07 (–1.38, 0.70)	–0.47 (–1.12, 1.45)
Self-efficacy^a^	2	13	9 (0.13%)	–0.36 (–0.73, –0.12)	–0.47 (–1.11, 0.18)
Monitoring	1	7	7 (0.18%)	–0.43 (–0.82, 0.21)	–0.33 (–0.89, 0.20)
Learning strategies	2	14	11 (0.15%)	–0.32 (–0.72, 0.88)	–0.52 (–1.04, –0.08)
Motivational strategies	1	4	4 (0.18%)	–0.14 (–0.28, 0.05)	–0.94 (–1.09, –0.76)
Persistence	1	6	2 (0.06%)	–0.41 (–0.67, –0.21)	–0.24 (–0.44, –0.01)
Self-evaluation	2	7	1 (0.03%)	–0.21 (–0.95, 0.26)	–0.55 (–0.94, 0.44)

*We also calculated Mardia’s (1970) measures of multivariate skewness and kurtosis for each CP-SRLI component. The measures indicated non-normal multivariate distributions for all components except for Motivational strategies.*

*^a^Self-Efficacy, Self-Efficacy for Self-Regulated Learning.*

#### Criterion Measures

To assess criterion validity, we used several simple self-report self-regulation variables reflected in behavior (time management of daily activities and schoolwork, and regularity of schoolwork), school performance variables, and a more complex externally assessed metacognitive knowledge task. The selection of variables was based on (meta-analytic) research on the positive association of school performance with time management, regularity of schoolwork (Cyril, 2015; Núñez et al., 2015; Aeon et al., 2021), and metacognitive knowledge (Gomes et al., 2014; Ergen and Kanadli, 2017; Stephanou and Mpiontini, 2017; Simons et al., 2020).

We recorded average school grade in the previous year, binary-coded involvement in school and out-of-school extracurricular activities, and responses to single-item measures (all with 5-point Likert-type scales) regarding time management of daily activities, schoolwork, learning for a test, and regularity of studying. More detailed descriptions of these single-item measures are provided to the notes of Table 2.

**TABLE 2:** Descriptive statistics and McDonald’s omegas for the CP-SRLI components, and descriptive statistics for the criterion measures.

Measures	*M*	*SD*	Skewness	Kurtosis	ω
**CP-SRLI components**					
Task orientation	3.26	0.71	–0.20	–0.13	0.69
Planning	3.65	0.75	–0.36	0.24	0.53
Motivation					
Extrinsic regulation	2.41	1.10	0.42	–0.68	0.87
Introjected regulation	3.07	0.97	–0.11	–0.56	0.75
Identified regulation	4.05	0.89	–1.00	0.75	0.82
Intrinsic regulation	2.52	0.96	0.31	–0.67	0.88
Self-efficacy					
Self-efficacy regulation	3.46	0.72	–0.35	0.42	0.82
Self-efficacy motivation	3.42	0.93	–0.32	–0.20	0.87
Self-efficacy (one factor)	3.45	0.73	–0.36	0.42	0.89
Monitoring	3.46	0.70	–0.54	0.82	0.75
Learning strategies					
Elaboration	3.28	1.00	–0.31	–0.52	0.75
Rehearsal	3.55	0.98	–0.50	–0.23	0.73
Motivational strategies	3.16	0.86	–0.16	–0.15	0.64
Persistence	3.52	0.82	–0.46	0.01	0.87
Self-evaluation					
Self-evaluation: product	3.72	0.93	–0.66	0.01	0.79
Self-evaluation: process	2.75	0.96	0.00	–0.67	0.83
**Criterion measures**					
MCK score^a^	2.52	1.26	0.14	0.21	
Average school grade^b^	4.05	0.70	–0.45	–0.85	
School extra activities^c^	0.39	0.49			
Out-of-school extra activities^c^	0.70	0.46			
Time management of daily activities^d^	3.33	0.68	–0.27	–0.03	
Lack of time when learning for a test^e^	2.71	1.08	0.14	–0.39	
Time management of school work^f^	3.83	1.11	–0.71	–0.07	
I study regularly^g^	2.93	1.13	–0.05	–0.59	

*^a^MCK score, meta-cognitive knowledge score.*

*^b^Average school grade in previous year (possible range = 1–5).*

*^c^School and out-of-school extracurricular activities were binary coded (0 = does not participate in any activity, 1 = participates in at least one activity), so the arithmetic mean shows a proportion of pupils that were involved in at least one activity.*

*^d^In spite of all school and out-of-school extracurricular activities, I am left with enough time for studying and doing homework. (possible range = 1–5).*

*^e^I frequently run out of time when studying for a test. (possible range = 1–5).*

*^f^When faced with a difficult or time-consuming school assignment, I organize my time in such way that I can complete the work on time. (possible range = 1–5).*

*^g^I study and do my homework regularly. (possible range = 1–5).*

We also measured students’ metacognitive knowledge with an open-ended task. The theoretical background, the analysis of the students’ responses, and the procedure for calculating the global metacognitive score (MCK score) are described in section “Deriving the Metacognitive Knowledge Score (MCK score).”

#### Translation of the Children’s Perceived Use of Self-Regulated Learning Inventory

We employed a thorough multi-stage translation with back-translations from Slovene to English and Dutch, and multiple independent corrections and verifications [the translation was done according to the guidelines of the International Test Commission, 2018]. The translation team included Slovene educational psychologists, professional English and Dutch translators, and the original authors of the CP-SRLI.

#### Procedure

After approval by the Ethics Committee of the Faculty of Arts, University of Ljubljana, we conducted the study in the classrooms of the selected primary schools. Only children whose parents provided signed informed consent participated in the study. Test materials were applied in the paper-pencil form.

Data on criterion measures, i.e., average school grade in the previous year (which served as a proxy for academic achievement), time management variables, and metacognitive knowledge score (see section “Criterion Measures”) were collected from different subsamples as logistical circumstances (limited time in classrooms) allowed us to administer only the CP-SRLI in the first part of the data acquisition process (this validation study was a part of a larger research project that was carried out in several steps). Thus, the CP-SRLI data and final school grades in the previous year were collected from all participants (*n* = 541). In the second part of the data acquisition process, data on time management variables was gathered from 314 students. Of the latter subsample (i.e., *n* = 314), 155 students participated in the metacognitive knowledge task (MCK task), while the remaining students were involved in other project tasks that were not relevant to this validation study.

##### Deriving the Metacognitive Knowledge Score

To assess students’ metacognitive knowledge, they briefly reviewed an interactive electronic chemistry lesson on essential oils. We then asked them to provide a written description of all stages of the learning process (before, during, and after the lesson) that would, in their opinion, lead to excellent knowledge of the topic. The schema for coding the descriptions was developed according to contemporary learning theories and classification procedures (McInerney, 2013), and consisted of nine categories of metacognitive knowledge. The first group of categories referred to declarative knowledge and included metacognitive knowledge about oneself (e.g., “I learn better when no one disturbs me”) and about the task (e.g., the student knows how challenging the task is and what a good solution for a particular task is). The second group of categories was related to procedural knowledge, i.e., the student is aware of how to apply what they know. This group was divided into cognitive and metacognitive strategies. The cognitive strategies included rehearsal (e.g., the student knows that it is useful to repeat the material until they remember everything), elaboration (e.g., to take notes while reading), and organization (e.g., to write things down on cards). The metacognitive strategies that represent control of the learning process were as follows: task-related planning (e.g., dividing the material into small parts, activating prior knowledge), self-referential planning (e.g., setting up the learning space, meeting physiological needs, preparing tools), task-related monitoring and regulation (e.g., doing some tasks and returning to the parts where the tasks did not go well after learning was completed), self-referential monitoring and regulation (e.g., effort management, concentration, breaks), and evaluation (at the end of learning, the students determine whether the goal was achieved). The responses were analyzed and binary coded (i.e., 0 = none, 1 = at least one mention of a particular category in the student’s description of the learning process) by two authors (educational psychologists). The binary scores were summed to produce the global metacognitive knowledge (MCK) score, with a higher score indicating better metacognitive knowledge about the SRL process.

#### Statistical Analyses

All statistical analyses were done in R (R Core Team, 2019). Single- and multiple-group confirmatory factor analyses were conducted with *lavaan* package (Rosseel, 2012). The following cutoff values of fit indices were considered as indicating acceptable fit of the tested models (Marsh et al., 2004): CFI ≥ 0.90, RMSEA and SRMR ≤ 0.08. Following the procedure of the original CP-SRLI authors, we carried out separate CFAs for each scale. Since most item distributions deviated from the normal distribution (Table 1), we used the robust maximum likelihood estimator (MLM—maximum likelihood parameter estimation with standard errors and a mean-adjusted chi-square test statistic that are robust to non-normality). The scales of the latent factors were identified by fixing their variance to unity in all examined models. All CP-SRLI items contained a very small number of missing values (Table 1). We conducted the non-parametric missing value imputation with the missForest package (Stekhoven and Buehlmann, 2012) by using the missForest function with the default argument values, i.e., maximum number of iterations set to 10 and number of trees to grow in each forest set to 100.

Exploratory factor analysis was conducted with the *psych* R package (Revelle, 2018) using the minimal residual extraction method and *oblimin* rotation.

We calculated McDonald’s ω_t_ coefficients (i.e., omega total) as reliability estimates of the CP-SRLI (sub) components scores.

## Results and Discussion

### Internal Structure Validity and Reliability of the Children’s Perceived Use of Self-Regulated Learning Inventory Components

Internal structure validity of the CP-SRLI components was assessed by single-group confirmatory factor analyses. Model fit was satisfactory for Task Orientation, Planning, Motivational Strategies, Monitoring, Persistence, and Self-Evaluation (Table 3), while the fit of other components (Motivation, Self-Efficacy, and Learning Strategies) required further inspection. The items (and their factor-related abbreviations), their standardized loadings, and descriptive statistics are presented in Supplementary Table 1, and the correlations between the CP-SRLI scale scores are presented in Supplementary Table 2.

**TABLE 3:** Fit indices for the CP-SRLI components.

Model	S-B χ^2^	*df*	*P*	CFI	RMSEA	RMSEA (90% CI)	SRMR
Task orientation	31.4	9	<0.001	0.943	0.068	0.045–0.093	0.046
Planning	7.9	2	0.019	0.953	0.074	0.027–0.128	0.033
Motivation	350.5	71	<0.001	0.911	0.085	0.077–0.094	0.078
Motivation mod1	308.0	71	<0.001	0.925	0.079	0.070–0.089	0.073
Motivation mod2	239.1	59	<0.001	0.937	0.075	0.066–0.084	0.066
Self-efficacy^a^	279.2	64	<0.001	0.901	0.079	0.071–0.087	0.064
Self-efficacy mod1	202.1	63	<0.001	0.936	0.064	0.056–0.072	0.054
Self-efficacy 1-factor	340.6	65	<0.001	0.874	0.089	0.081–0.096	0.072
Self-efficacy 1-factor mod	252.5	64	<0.001	0.914	0.074	0.066–0.082	0.062
Self-efficacy (bifactor model)	135.4	52	<0.001	0.960	0.054	0.045–0.064	0.035
Monitoring	45.9	14	<0.001	0.934	0.065	0.047–0.083	0.046
Learning strategies	319.1	76	<0.001	0.834	0.077	0.069–0.085	0.071
Learning strategies EFA (2 factors)^b^	4.71	4	0.318	/	0.019	0.000–0.070	0.010
Motivational strategies	7.2	2	0.027	0.978	0.069	0.026–0.120	0.035
Persistence	25.1	9	0.003	0.985	0.057	0.035–0.081	0.025
Self-evaluation	41.4	13	<0.001	0.978	0.064	0.044–0.084	0.040

*S-B χ^2^, Satorra-Bentler χ^2^; mod, modified.*

*Motivation mod1, IDR1 item from Identified Regulation moved to Intrinsic Regulation; Motivation mod2, IDR1 item excluded from Identified Regulation; Self-Efficacy mod1, correlated residuals of items SER5 and SER7; Self-Efficacy 1-factor mod, 1-factor model with correlated residuals of items SER5 and SER7.*

*^a^Self-Efficacy, Self-Efficacy for Self-Regulated Learning.*

*^b^TLI, 0.996.*

For Motivation, the largest modification index (107.0, accounting for almost a third of the model chi-square) suggested that the first Identified Regulation item (“… because I want to learn new things”) should be an indicator of Intrinsic Regulation instead of Identified Regulation. This seems a logical result; in our opinion, the formulation “want to learn” implies interest and enjoyment, and thus corresponds more to the concept of intrinsic motivation as defined by Ryan and Deci (2000) than to the concept of identified regulation. Moving this item to Intrinsic Motivation scale improved the model fit substantially. However, removing this item from Identified Regulation resulted in an even slightly better fit and reliability of this scale (Table 2; omega for Intrinsic Regulation changed from 0.88 to 0.89). The correlations between the components (Supplementary Table 2) followed the expected pattern and were highly comparable with that of Vandevelde et al. (2013).

Modification indices for the two-factor Self-Efficacy model showed that the correlated residuals of two Regulation items (SER5 and SER7) were the largest source of misfit. Both items have quite similar content that pertains to finding and summarizing key information, which is the most probable cause for correlated residuals of these two items. Estimating the correlation between their residuals significantly improved model fit. As noted by Vandevelde et al. (2013), self-efficacy was presumed to be a unidimensional construct, but they tested only the two-factor model. Since the correlation between Regulation and Motivation factors was 0.87 (0.83 in the study by Vandevelde et al.), we additionally tested the fit of the one-factor model and a bifactor model with a general factor and two uncorrelated specific factors (i.e., Self-Efficacy Regulation and Self-Efficacy Motivation; see Dunn and McCray, 2020, for the advantages of bifactor models compared to higher-order ones). After allowing the residuals of SER5 and SER7 in the one-factor model to be correlated (this parameter was again associated with the largest MI, i.e., 111.9), the model fit became acceptable. Concerning the bifactor model, the fit to the data was adequate and the loadings on the general factor were moderate to high (see Supplementary Table 1). The results of the one-factor and the bifactor models provided empirical support for the calculation of the total Self-Efficacy score. This score had high reliability (Table 2).

The fit of the two-factor model for Learning Strategies proposed by Vandevelde et al. (2013) was inadequate. The modification indices consisted of many comparable suggestions for correlated item residuals and cross-loadings, so it was not possible to make any theoretically sound modifications. We therefore conducted exploratory factor analysis. Parallel analysis indicated that two factors should be extracted. To get a simple and stable solution, we retained items with high loadings, i.e., higher than 0.60 (Garson, 2013), and low complexity (Hofmann, 1978). The initial solution obtained with all items (Supplementary Table 3; LDL stands for deep-level and LSL for surface learning strategies) was unsatisfactory due to low loadings of the excluded items, cross-loadings (LDL1, LDL3, LDL6), and a lower loading on the respective factor (LDL6, LSL2). The second analysis with the retained six items showed a simple and interpretable two-factor structure accounting for 49% of the variance. This model had an excellent fit (Table 3); according to the content of their items, the factors were named Elaboration (i.e., identifying and summarizing key information) and Rehearsal. One possible reason for this difference between the original and our solution is that by the age of 14–15 years, students have already gained experience with various strategies they had learned in the lower grades, and retained only those that were particularly relevant to them and proved to be effective in achieving their learning goals. Assessment in Slovene schools focuses primarily on memorization and comprehension, so the strategies of rehearsal and elaboration have proven to be the most successful dimensions of SRL.

McDonald’s omegas total (Table 2) were similar to reliability coefficients estimated by Vandevelde et al. (2013), indicating acceptable or good reliability for all components, except for Motivational Strategies and (especially) Planning, which had poor internal consistency.

### Measurement Invariance Across Gender

We tested hypotheses regarding equality of factor loadings (metric invariance) and equality of item intercepts (scalar invariance) across gender for each CP-SRLI component. Comparisons of nested models were done via the likelihood ratio test (*p* ≤ 0.05).

Full metric invariance was found for all (sub) components, except for Learning Strategies (Supplementary Table 4). After reviewing the modification indices and freeing the loading of item LSL3, the chi-square test of difference between the configural and partial metric invariance model was no longer statistically significant. Full scalar invariance was observed for Planning, Learning Strategies, and Self-Evaluation. We also did not find any non-invariant item intercepts in External Regulation (the subcomponent of Motivation). Partial scalar invariance that could still allow meaningful factor means comparisons was found for Monitoring (we had to estimate one out of seven intercepts), Motivational Strategies (one out of four intercepts), Persistence (one out of six intercepts), Identified Regulation (one out of three intercepts), and Task Orientation (two out of six intercepts). Partial scalar invariance of other components (Self-Efficacy and two subscales of Motivation) was not supported—half or more intercepts should be freed to achieve a non-significant change between the metric and scalar models.

### Criterion Validity

The correlations between the CP-SRLI components and selected criterion measures followed the expected pattern (Table 4). Externally assessed metacognitive knowledge showed low to moderate positive correlations with all components (except for a low negative correlation with Extrinsic Regulation), and was most strongly associated with Planning, Self-Efficacy, Monitoring, and Persistence. These results show that metacognitive knowledge is a prerequisite for the use of metacognitive strategies and regulation of motivation. Neurological changes in early adolescence, such as synaptic pruning and myelination of axons in prefrontal cortex, and gradual increase of connections between the prefrontal cortex, the limbic system, and other parts of the brain enable more efficient information processing. These changes improve the abilities of logical reasoning, information integration, long-term planning, and cognitive and emotional self-regulation (Berk, 2012). As predicted, our results showed that adolescents’ ability to gain insight into how they function as learners while considering the task requirements (metacognitive knowledge) leads to higher use of (meta) cognitive strategies in concrete learning situations, better developed motivational strategies, and better regulation of self-efficacy in learning.

**TABLE 4:** Intercorrelations among the criterion measures, and their correlations with the CP-SRLI components.

	1	2	3	4	5	6	7	8
**Criterion measures**								
1. MCK score								
2. Average school grade	0.33							
3. School extra activities	0.10	0.22						
4. Out-of-school extra activities	0.16	0.23	0.12					
5. Time management of daily activities	0.28	0.26	0.10	0.08				
6. Lack of time when learning for a test	–0.06	–0.29	–0.07	–0.01	–0.32			
7. Time management of school work	0.25	0.26	0.06	0.10	0.63	–0.26		
8. I study regularly	0.21	0.09	0.09	0.03	0.75	–0.24	0.36	
**CP-SRLI components**								
Task orientation	0.15	0.08	0.14	0.07	0.34	–0.03	0.18	0.28
Planning	0.37	0.27	0.10	0.11	0.47	–0.08	0.38	0.37
Motivation								
Extrinsic regulation	–0.11	–0.14	0.01	–0.02	–0.18	0.24	–0.14	–0.12
Introjected regulation	0.23	0.10	0.13	0.02	0.32	0.01	0.22	0.23
Identified regulation	0.26	0.26	0.11	0.07	0.43	–0.22	0.37	0.33
Intrinsic regulation	0.28	0.21	0.17	0.11	0.47	–0.29	0.19	0.45
Self-efficacy								
Regulation	0.37	0.35	0.13	0.16	0.47	–0.23	0.38	0.39
Motivation	0.34	0.30	0.11	0.13	0.53	–0.29	0.38	0.48
Monitoring	0.31	0.24	0.06	0.08	0.34	0.03	0.24	0.25
Learning strategies								
Elaboration	0.22	0.19	–0.01	0.11	0.18	0.06	0.18	0.18
Rehearsal	0.13	0.11	–0.01	–0.01	0.17	0.02	0.19	0.06
Motivational strategies	0.17	0.09	0.05	–0.03	0.22	0.16	0.15	0.16
Persistence	0.36	0.30	0.10	0.09	0.56	–0.28	0.43	0.48
Self-evaluation								
Product	0.28	0.13	0.03	0.08	0.36	–0.11	0.29	0.30
Process	0.03	–0.10	0.08	0.02	0.19	0.05	0.08	0.23

*MCK score, metacognitive knowledge score. See notes to Table 2 for more detailed descriptions of the single-item criterion measures. All correlations larger than | 0.15| are statistically significant at the 5% significance level.*

Similar, but slightly lower correlations were observed between the CP-SRLI components and the average school grade in the previous year. These lower correlations may be explained by the fact that school grades depend on many factors other than students’ self-regulation abilities, such as intelligence (e.g., Roth et al., 2015), working memory and logical reasoning (e.g., Krumm et al., 2008), attention (e.g., Metallidou et al., 2016), self-belief, interest, affect (Efklides, 2011), and gender (Lekholm and Cliffordson, 2009).

Participation in school- and out-of-school extracurricular activities did not seem to correlate saliently with the SRL components, so these variables may not be the best indicators of students’ SRL skills. Even though we expected that successful management of (many different) extracurricular activities would be characteristic of students with adequate SRL competences, the mere presence/absence of such activities may not carry sufficient information about students’ engagement. The exact type and number of such activities, as well as time spent on them may be more informative and should be included in future studies to further investigate the relationship between students’ ability to regulate their in-school and out-of-school activities.

Highly intercorrelated time- and workload-management measures (i.e., time management of daily and schoolwork activities, lack of time when studying for a test, and regular studying) showed a similar pattern of correlations with the CP-SRLI components. The strongest positive correlations of these measures were found with Planning, Intrinsic Motivation, the most autonomous form of extrinsic motivation (i.e., Identified Regulation), Self-Efficacy, Monitoring, Persistence, and Product Self-Evaluation. These correlations suggest that time management skills are key to regulating all school-related activities (McInerney, 2013; Cyril, 2015), e.g., homework, learning assignments, and different school projects.

## Conclusion

The Slovenian version of the CP-SRLI showed acceptable construct validity and reliability, but some modifications were necessary. First, the original Learning Strategies factors were simplified into more narrow factors, Elaboration and Rehearsal. However, this two-factor solution should be validated on an independent sample in future studies. Second, we provided support for a total scale score on Self-Efficacy, as originally hypothesized by Vandevelde et al. (2013). Third, we proposed moving one item from the Identified Regulation to the Intrinsic Regulation subscale. Regarding measurement invariance, Self-Efficacy was the most problematic component; at the same absolute level of self-efficacy, girls are expected to have a higher response than boys on all Self-Efficacy items. This could partly be attributed to the gender gap in perceived academic effort, investment, and study culture (e.g., Houtte, 2004), which could influence the way girls and boys perceive and respond to the Self-Efficacy items. While this issue should be further investigated (e.g., with cognitive interviews to identify the possible causes of differential item functioning), the other scales of the CP-SRLI can be used for means comparisons by gender without reservation. With the mentioned adjustments, the Slovenian version of the CP-SRLI proved to be a sufficiently valid and reliable instrument for assessing children’s learning self-regulation. The self-regulation skills assessed by the CP-SRLI are not only reflected in school work that students carry out at home, but may also indicate how well students regulate their activities while in school. Thus, the scores obtained by the CP-SRLI may help school professionals plan and evaluate interventions for developing students’ self-regulation skills.

## Data Availability Statement

The datasets presented in this study can be found in online repositories. The names of the repository/repositories and accession number(s) can be found below: https://osf.io/73jkv/; Open Science Framework (OSF).

## Ethics Statement

The studies involving human participants were reviewed and approved by the Ethics Commission of Faculty of Arts, University of Ljubljana. Written informed consent to participate in this study was provided by the participants’ legal guardian/next of kin.

## Author Contributions

LK, AP, and CP conceived the idea for the study. KD, CP, SP, and TP carried out all tasks for the translation of the CP-SRLI to Slovenian with the help of all other authors. Data acquisition was done by BB, CB, AG, KD, AK, ŽL, MM, MP, and CP. LK analyzed the data with the help of AP. LK wrote the manuscript with the support of AP, TP, SP, and CP, while all other authors commented on the manuscript. All authors contributed to the article and approved the submitted version.

## Conflict of Interest

The authors declare that the research was conducted in the absence of any commercial or financial relationships that could be construed as a potential conflict of interest.

## Publisher’s Note

All claims expressed in this article are solely those of the authors and do not necessarily represent those of their affiliated organizations, or those of the publisher, the editors and the reviewers. Any product that may be evaluated in this article, or claim that may be made by its manufacturer, is not guaranteed or endorsed by the publisher.
