# Supplementary material for: Slovenian Validation of the Children’s Perceived Use of Self-Regulated Learning Inventory
Source: Front Psychol. 2022 Jan 14;12:730386. doi: 10.3389/fpsyg.2021.730386 (PMC8795087; doi:10.3389/fpsyg.2021.730386)
Supplement: Supplementary file 1 [file Table_1.DOCX]

Supplementary Material

Slovenian Validation of the Children's Perceived use of Self-Regulated Learning Inventory (CP-SRLI)

**Luka Komidar, Anja Podlesek, Tina Pirc, Sonja Pečjak, Katja Depolli Steiner, Melita Puklek Levpušček, Alenka Gril, Bojana Boh Podgornik, Aleš Hladnik, Alenka Kavčič, Ciril Bohak, Žiga Lesar, Matija Marolt, Matevž Pesek, Cirila Peklaj**

*** Correspondence:**

Luka Komidar:

luka.komidar@ff.uni-lj.si

# Supplementary Tables

**Table S1.** *Standardized Loadings, Obtained by the CFAs, and Item Descriptive Statistics for the CP-SRLI*

|  | **CP-SRLI Components and Items** | λ | *M* | *SD* | Skew | Kurt |
| --- | --- | --- | --- | --- | --- | --- |
|  | **Task Orientation** |  |  |  |  |  |
| TO1 | Before I start my schoolwork, I read the instructions carefully. | .41 | 3.84 | 0.86 | –0.45 | 0.07 |
| TO2 | Before I start my schoolwork, I ask myself: ‘What is it about? What do I already know about it?’ | .71 | 3.30 | 1.11 | –0.28 | –0.54 |
| TO3 | Before I start my schoolwork, I ask myself: ‘Do I know what kind of a task this is? | .63 | 3.01 | 1.17 | –0.11 | –0.86 |
| TO4 | If I get a task similar to one I have already done, I ask myself: ‘How did I approach it last time? Was that a good approach?’ | .50 | 3.66 | 1.09 | –0.78 | 0.14 |
| TO5 | Before I start my schoolwork, I ask myself: ‘What do I feel about this task (fun, difficult, interesting, ...)?’ | .49 | 2.72 | 1.28 | 0.22 | –1.02 |
| TO6 | Before I start my schoolwork, I ask myself: ‘Will I succeed?’ | .33 | 3.02 | 1.32 | –0.02 | –1.08 |
|  | **Planning** |  |  |  |  |  |
| PL2 | Before I start my schoolwork, I decide what to do first and what later. | .63 | 4.00 | 1.11 | –0.95 | 0.12 |
| PL3 | If I find my schoolwork difficult, I allow more time for it. | .44 | 3.88 | 1.07 | –0.81 | 0.05 |
| PL4 | If I have to do a large assignment, I start some days before and every day I do a piece of it. | .40 | 2.96 | 1.34 | 0.07 | –1.12 |
| PL5 | Before I start my schoolwork, I think how much time I will need. | .41 | 3.74 | 1.16 | –0.67 | –0.42 |
|  | **Motivation (I do my best for school, …)** |  |  |  |  |  |
|  | *Extrinsic Regulation* |  |  |  |  |  |
| ER1 | because I am supposed to do so by others (my parents, the teacher, etc.). | .83 | 2.50 | 1.25 | 0.42 | –0.82 |
| ER2 | because others (my parents, the teacher, etc.) oblige me to do so. | .90 | 2.51 | 1.24 | 0.37 | –0.86 |
| ER3 | because others (my parents, the teacher, etc.) force me to do so. | .76 | 2.20 | 1.21 | 0.70 | –0.52 |
|  | *Introjected Regulation* |  |  |  |  |  |
| INR1 | because I would feel guilty if I didn’t do my best. | .59 | 3.14 | 1.33 | –0.12 | –1.12 |
| INR2 | because I would feel ashamed if I didn’t do my best. | .64 | 3.02 | 1.31 | –0.11 | –1.04 |
| INR3 | because I want others (my parents, the teacher, etc.) to think I’m smart. | .51 | 2.65 | 1.32 | 0.30 | –1.06 |
| INR4 | because I want to show others (my parents, the teacher, etc.) that I am a good student. | .62 | 3.47 | 1.19 | –0.44 | –0.69 |
|  | *Identified Regulation* |  |  |  |  |  |
| IDR2 | because I think it is important for the future. | .74 | 4.28 | 0.97 | –1.38 | 1.45 |
| IDR3 | because I find it useful for myself. | .83 | 3.90 | 1.09 | –0.91 | 0.30 |
| IDR4 | because I find it important to me as a person. | .77 | 3.98 | 1.04 | –0.91 | 0.30 |
|  | *Intrinsic Regulation* |  |  |  |  |  |
| IDR1 | because I want to learn new things. | .67 | 3.34 | 1.15 | –0.32 | –0.50 |
| IM1 | because I find it very interesting. | .83 | 2.40 | 1.14 | 0.33 | –0.81 |
| IM2 | because I like doing it. | .88 | 2.27 | 1.15 | 0.47 | –0.71 |
| IM3 | because I enjoy doing it. | .84 | 2.07 | 1.08 | 0.65 | –0.50 |
|  | **Self-Efficacy for Self-Regulated Learning: 2 factors (I'm good at …)** |  |  |  |  |  |
|  | *Regulation* |  |  |  |  |  |
| SER1 | thinking at first about how I will approach my schoolwork. | .63 | 3.34 | 1.15 | –0.24 | –0.63 |
| SER2 | planning the timing of my schoolwork before I start making it. | .62 | 3.52 | 1.17 | –0.39 | –0.74 |
| SER3 | working with consistent attention during my schoolwork. | .61 | 3.20 | 1.10 | –0.22 | –0.44 |
| SER4 | knowing what is important and less important when studying. | .59 | 3.75 | 1.01 | –0.73 | 0.18 |
| SER5 | pointing out the information that is important when studying. | .48 | 3.74 | 1.09 | –0.65 | –0.22 |
| SER6 | connecting new things to what I already know. | .49 | 3.52 | 1.05 | –0.39 | –0.36 |
| SER7 | making a scheme or mind map when studying. | .37 | 3.21 | 1.32 | –0.12 | –1.11 |
| SER8 | changing my strategy when it doesn’t work out during my schoolwork. | .57 | 3.25 | 1.06 | –0.14 | –0.58 |
| SER9 | checking my schoolwork by myself. | .74 | 3.59 | 1.09 | –0.50 | –0.33 |
|  | *Motivation* |  |  |  |  |  |
| SEM1 | motivating myself to start making on my schoolwork. | .73 | 3.15 | 1.16 | –0.16 | –0.70 |
| SEM2 | motivating myself to finish my schoolwork. | .80 | 3.39 | 1.08 | –0.31 | –0.39 |
| SEM3 | making my schoolwork, even if I find it boring or difficult. | .77 | 3.55 | 1.08 | –0.45 | –0.40 |
| SEM4 | holding onto my schoolwork. | .84 | 3.58 | 1.09 | –0.44 | –0.38 |
|  | **Self-Efficacy for Self-Regulated Learning: 1 factor (loadings on the general factor from the bifactor model)** |  |  |  |  |  |
| SER1 | thinking at first about how I will approach my schoolwork. | .55 |  |  |  |  |
| SER2 | planning the timing of my schoolwork before I start making it. | .58 |  |  |  |  |
| SER3 | working with consistent attention during my schoolwork. | .67 |  |  |  |  |
| SER4 | knowing what is important and less important when studying. | .50 |  |  |  |  |
| SER5 | pointing out the information that is important when studying. | .35 |  |  |  |  |
| SER6 | connecting new things to what I already know. | .42 |  |  |  |  |
| SER7 | making a scheme or mind map when studying. | .27 |  |  |  |  |
| SER8 | changing my strategy when it doesn’t work out during my schoolwork. | .49 |  |  |  |  |
| SER9 | checking my schoolwork by myself. | .70 |  |  |  |  |
| SEM1 | motivating myself to start making on my schoolwork. | .75 |  |  |  |  |
| SEM2 | motivating myself to finish my schoolwork. | .79 |  |  |  |  |
| SEM3 | making my schoolwork, even if I find it boring or difficult. | .73 |  |  |  |  |
| SEM4 | holding onto my schoolwork. | .79 |  |  |  |  |
|  | **Monitoring** |  |  |  |  |  |
| MT1 | During my schoolwork, I ask myself: ‘Is it working well in this way? | .43 | 2.81 | 1.22 | 0.21 | –0.89 |
| MT2 | If I notice something isn’t working out, I try a different approach. | .54 | 3.46 | 1.02 | –0.44 | –0.14 |
| MT3 | During my schoolwork, I ask myself: ‘Do I still understand everything?’ | .61 | 3.24 | 1.11 | –0.22 | –0.56 |
| MT4 | During my schoolwork, I ask myself: ‘Do I still have enough time? | .52 | 3.49 | 1.23 | –0.45 | –0.71 |
| MT5 | During my schoolwork, I check what I already have done from time to time and how much I still have to do. | .53 | 3.84 | 1.08 | –0.79 | 0.03 |
| MT6 | During my schoolwork, I follow my plan. | .49 | 3.62 | 1.07 | –0.49 | –0.25 |
| MT7 | During my schoolwork, I ask myself: ‘What part is difficult? What do I have to practice some more?’ | .67 | 3.75 | 1.08 | –0.82 | 0.20 |
|  | **Learning Strategies: 2 factors, obtained by EFA, see Table S3 for standardized loadings** |  |  |  |  |  |
|  | *Deep-level learning strategies* => *Elaboration* |  |  |  |  |  |
| LDL1 | I try to repeat the new material in my own words. | excluded | 3.74 | 1.10 | –0.70 | –0.08 |
| LDL2 | I make a summary. |  | 3.43 | 1.22 | –0.33 | –0.81 |
| LDL3 | I use tricks or mnemonics to remember something easier. | excluded | 3.27 | 1.24 | –0.25 | –0.87 |
| LDL4 | I link it to what I already know. | excluded | 3.62 | 1.06 | –0.55 | –0.17 |
| LDL5 | I look for examples connected to what I am learning. | excluded | 3.17 | 1.13 | –0.15 | –0.71 |
| LDL6 | I make up test questions and answer them after studying. | excluded | 3.34 | 1.23 | –0.28 | –0.85 |
| LDL7 | I make a scheme or a mind map. |  | 2.68 | 1.31 | 0.28 | –1.04 |
| LDL8 | I mark important information or write it down. |  | 3.74 | 1.20 | –0.72 | –0.35 |
| LDL9 | I look for the main subjects or topics. | excluded | 3.41 | 1.11 | –0.51 | –0.32 |
| LDL10 | I figure out the meaning of difficult words. | excluded | 3.62 | 1.05 | –0.52 | –0.24 |
|  | *Surface-level learning strategies* => *Rehearsal* |  |  |  |  |  |
| LSL1 | I read or recall everything again and again until I know it by heart. |  | 3.41 | 1.28 | –0.39 | –0.86 |
| LSL2 | I copy everything until I know it by heart. | excluded | 2.04 | 1.14 | 0.88 | –0.15 |
| LSL3 | I cover up part of the material and try to say it out loud. |  | 3.58 | 1.24 | –0.61 | –0.57 |
| LSL4 | I practice until I know everything. |  | 3.66 | 1.12 | –0.61 | –0.29 |
|  | **Motivational Strategies^a^** |  |  |  |  |  |
| MOTS1 | During my schoolwork, I motivate myself to keep working. | .65 | 3.28 | 1.20 | –0.23 | –0.76 |
| MOTS4 | During my schoolwork, I say to myself: ‘Just a little more and it is finished!’ | .37 | 3.36 | 1.33 | –0.28 | –1.07 |
| MOTS5 | During my schoolwork, I say to myself: ‘You can do it, just keep on working!’ | .80 | 2.93 | 1.30 | 0.05 | –1.09 |
| MOTS6 | During my schoolwork, I think about reasons why it is important to complete this schoolwork. | .35 | 3.08 | 1.20 | –0.11 | –0.85 |
|  | **Persistence** |  |  |  |  |  |
| P1 | Even if I would rather do other things, I make myself start my schoolwork. | .77 | 3.12 | 1.11 | –0.21 | –0.44 |
| P2 | Even if my schoolwork is difficult or boring, I do my best. | .76 | 3.83 | 1.04 | –0.67 | –0.11 |
| P3 | Even if I would rather do other things, I finish my schoolwork. | .78 | 3.36 | 1.10 | –0.32 | –0.39 |
| P4 | I carry on until I finish my schoolwork. | .78 | 3.57 | 1.03 | –0.31 | –0.39 |
| P5 | During my schoolwork, I work attentively and don’t take my mind off it. | .66 | 3.62 | 0.97 | –0.41 | –0.01 |
| P6 | If I am distracted while doing my schoolwork, I immediately try to continue working. | .63 | 3.61 | 1.04 | –0.53 | –0.13 |
|  | **Self-Evaluation (After finishing my schoolwork, …)** |  |  |  |  |  |
|  | *Product Evaluation* |  |  |  |  |  |
| SPROD1 | I go over my answers again. | .65 | 3.35 | 1.21 | –0.32 | –0.83 |
| SPROD2 | I check that I haven’t forgotten anything. | .81 | 3.85 | 1.08 | –0.74 | –0.14 |
| SPROD3 | I check if I have done everything that was asked for. | .77 | 3.97 | 1.04 | –0.95 | 0.44 |
|  | *Proces Evaluation* |  |  |  |  |  |
| SPROC1 | I ask myself: ‘Have I done it the right way?’ | .78 | 2.82 | 1.15 | 0.07 | –0.77 |
| SPROC2 | I ask myself: ‘Will I use a similar approach next time, or should I choose a different approach?’ | .78 | 2.65 | 1.17 | 0.20 | –0.81 |
| SPROC3 | I ask myself: ‘Did that way of doing it worked well?’ | .84 | 2.91 | 1.18 | –0.01 | –0.83 |
| SPROC4 | I ask myself: ‘How did I feel about it? (fun, difficult, boring, interesting, ...)?’ | .54 | 2.62 | 1.26 | 0.26 | –0.93 |

The loadings in all tested models were statistically significant at the 0.1% (*p* < .001) significance level.

^a^ In case of motivational strategies, we used the abbreviations (MOTS1, 4, 5, 6) assigned to the items by Vandevelde et al. (2013).

**Table S2.** *Correlations Between the CP-SRLI Scale-Scores*

|  |  | 1 | 2 | 3 | 4 | 5 | 6 | 7 | 8 | 9 | 10 | 11 | 12 | 13 | 14 |
| --- | --- | --- | --- | --- | --- | --- | --- | --- | --- | --- | --- | --- | --- | --- | --- |
| 1 | Task Orientation |  |  |  |  |  |  |  |  |  |  |  |  |  |  |
| 2 | Planning | .49 |  |  |  |  |  |  |  |  |  |  |  |  |  |
|  | Motivation |  |  |  |  |  |  |  |  |  |  |  |  |  |  |
| 3 | Extrinsic Regulation | .00 | –.11 |  |  |  |  |  |  |  |  |  |  |  |  |
| 4 | Introjected Regulation | .34 | .31 | .20 |  |  |  |  |  |  |  |  |  |  |  |
| 5 | Identified Regulation | .34 | .44 | –.25 | .43 |  |  |  |  |  |  |  |  |  |  |
| 6 | Intrinsic Regulation | .43 | .34 | –.13 | .42 | .50 |  |  |  |  |  |  |  |  |  |
|  | Self-Efficacy |  |  |  |  |  |  |  |  |  |  |  |  |  |  |
| 7 | ... Regulation | .56 | .62 | –.17 | .43 | .56 | .51 |  |  |  |  |  |  |  |  |
| 8 | ... Motivation | .45 | .53 | –.23 | .40 | .58 | .53 | .70 |  |  |  |  |  |  |  |
| 9 | Monitoring | .65 | .58 | –.09 | .33 | .46 | .32 | .65 | .53 |  |  |  |  |  |  |
|  | Learning Strategies |  |  |  |  |  |  |  |  |  |  |  |  |  |  |
| 10 | … Elaboration | .26 | .38 | –.13 | .22 | .33 | .27 | .57 | .29 | .39 |  |  |  |  |  |
| 11 | … Repetition | .39 | .43 | –.11 | .24 | .38 | .27 | .42 | .43 | .49 | .25 |  |  |  |  |
| 12 | Motivational Strategies | .52 | .43 | –.01 | .34 | .31 | .29 | .46 | .43 | .68 | .31 | .40 |  |  |  |
| 13 | Persistence | .47 | .57 | –.25 | .36 | .61 | .49 | .67 | .82 | .56 | .32 | .48 | .42 |  |  |
|  | Self-Evaluation |  |  |  |  |  |  |  |  |  |  |  |  |  |  |
| 14 | … Product | .50 | .52 | –.06 | .43 | .47 | .39 | .67 | .56 | .54 | .39 | .44 | .41 | .58 |  |
| 15 | … Process | .61 | .39 | .03 | .41 | .31 | .42 | .55 | .44 | .62 | .35 | .35 | .58 | .42 | .49 |

*Note*. All correlations larger than |.09| are statistically significant at the significance level of 5%.

**Table S3.** *The Initial and Final Two-Factor Solutions for Learning Strategies Obtained by Exploratory Factor Analysis*

|  |  | All items | |  | Retained items | |
| --- | --- | --- | --- | --- | --- | --- |
|  | Items | LDL | LSL |  | Elab. | Reh. |
| LDL1 | I try to repeat the new material in my own words. | .35 | .33 |  |  |  |
| LDL2 | I make a summary. | .65 | .06 |  | .60 | .11 |
| LDL3 | I use tricks or mnemonics to remember something easier. | .40 | .26 |  |  |  |
| LDL4 | I link it to what I already know. | .42 | .19 |  |  |  |
| LDL5 | I look for examples connected to what I am learning. | .39 | .14 |  |  |  |
| LDL6 | I make up test questions and answer them after studying. | .21 | .44 |  |  |  |
| LDL7 | I make a scheme or a mind map. | .65 | –.14 |  | .63 | –.06 |
| LDL8 | I mark important information or write it down. | .75 | –.07 |  | .85 | –.01 |
| LDL9 | I look for the main subjects or topics. | .36 | .05 |  |  |  |
| LDL10 | I figure out the meaning of difficult words. | .43 | .18 |  |  |  |
| LSL1 | I read or recall everything again and again until I know it by heart. | –.07 | .75 |  | –.04 | .81 |
| LSL2 | I copy everything until I know it by heart. | .33 | .12 |  |  |  |
| LSL3 | I cover up part of the material and try to say it out loud. | .01 | .66 |  | .05 | .64 |
| LSL4 | I practice until I know everything. | .04 | .63 |  | .04 | .60 |
|  | Proportion of variance explained | .19 | .15 |  | .25 | .24 |

*Notes*. LDL = Deep-Level Learning Strategies, LSL = Surface Learning Strategies. Elab. = Elaboration (identifying and summarizing key information). Reh. = Rehearsal. Loadings of the retained items are printed on grey background.

**Table S4.** *Measurement Invariance Statistics for Gender Groups*

|  |  |  |  |  |  | Model difference test | | |
| --- | --- | --- | --- | --- | --- | --- | --- | --- |
|  | S-B χ^2^ | *df* | CFI | RMSEA | SRMR | Δχ^2^ | Δ*df* | *p* |
| Task orientation |  |  |  |  |  |  |  |  |
| Configural | 50.3 | 18 | .919 | .082 | .046 |  |  |  |
| Metric | 58.4 | 23 | .912 | .076 | .056 | 7.56 | 5 | .182 |
| Scalar | 88.6 | 28 | .849 | .090 | .069 | 33.72 | 5 | < .001 |
| Scalar (partial)^a^ | 63.9 | 26 | .905 | .074 | .058 | 5.32 | 3 | .150 |
| Planning |  |  |  |  |  |  |  |  |
| Configural | 14.38 | 4 | .898 | .098 | .031 |  |  |  |
| Metric | 19.20 | 7 | .880 | .080 | .043 | 4.72 | 3 | .193 |
| Scalar | 21.54 | 10 | .886 | .066 | .046 | 2.04 | 3 | .564 |
| Motivation |  |  |  |  |  |  |  |  |
| Configural | 291.2 | 138 | .951 | .064 | .066 |  |  |  |
| Metric | 303.4 | 148 | .950 | .062 | .071 | 11.41 | 10 | .327 |
| Scalar | 361.8 | 158 | .935 | .069 | .074 | 79.37 | 10 | < .001 |
| Scalar (partial)^b^ | 314.3 | 153 | .948 | .063 | .071 | 11.09 | 5 | .050 |
| Self-Efficacy |  |  |  |  |  |  |  |  |
| Configural | 258.5 | 126 | .936 | .062 | .056 |  |  |  |
| Metric | 276.5 | 137 | .932 | .061 | .063 | 15.78 | 11 | .149 |
| Scalar^c^ | 338.3 | 148 | .908 | .069 | .074 | 95.37 | 11 | < .001 |
| Monitoring |  |  |  |  |  |  |  |  |
| Configural | 57.0 | 26 | .929 | .067 | .045 |  |  |  |
| Metric | 63.1 | 32 | .929 | .060 | .050 | 4.13 | 6 | .659 |
| Scalar | 76.2 | 38 | .913 | .061 | .057 | 13.24 | 6 | .038 |
| Scalar (partial)^d^ | 67.5 | 37 | .930 | .055 | .052 | 3.11 | 5 | .683 |
| Learning Strategies |  |  |  |  |  |  |  |  |
| Configural | 21.8 | 16 | .989 | .037 | .036 |  |  |  |
| Metric | 35.0 | 20 | .971 | .053 | .049 | 13.57 | 4 | .009 |
| Metric (partial)^e^ | 25.3 | 19 | .988 | .035 | .041 | 3.46 | 3 | .325 |
| Scalar | 26.6 | 23 | .993 | .024 | .042 | 1.28 | 4 | .865 |
| Motivational Strategies |  |  |  |  |  |  |  |  |
| Configural | 8.8 | 4 | .977 | .066 | .032 |  |  |  |
| Metric | 10.1 | 7 | .985 | .040 | .035 | 1.19 | 3 | .754 |
| Scalar | 21.6 | 10 | .944 | .066 | .051 | 12.38 | 3 | .001 |
| Scalar (partial)^f^ | 14.3 | 9 | .975 | .047 | .040 | 4.33 | 2 | .115 |
| Persistence |  |  |  |  |  |  |  |  |
| Configural | 38.3 | 18 | .979 | .065 | .029 |  |  |  |
| Metric | 42.5 | 23 | .980 | .056 | .035 | 2.47 | 5 | .782 |
| Scalar | 57.4 | 28 | .970 | .062 | .043 | 16.45 | 5 | .006 |
| Scalar (partial)^g^ | 48.9 | 27 | .977 | .055 | .039 | 6.15 | 4 | .188 |
| Self-Evaluation |  |  |  |  |  |  |  |  |
| Configural | 60.3 | 26 | .973 | .070 | .041 |  |  |  |
| Metric | 71.3 | 31 | .968 | .069 | .050 | 10.88 | 5 | .054 |
| Scalar | 74.8 | 36 | .969 | .063 | .050 | 2.38 | 5 | .794 |

*Notes*. S-B χ^2^ = Satorra-Bentler scales (mean-adjusted) χ^2^.

See Table S1 in Supplementary materials for item contents and abbreviations.

^a^ Free intercepts of items TO1 and TO2.

^b^ Free intercepts of items INR1, INR3, IM1, IDR1 (a part of IM), and IDR2.

^c^ Achieving partial scalar invariance was not possible.

^d^ Free intercept of item MT5.

^e^ Free loading of item LSL3.

^f^ Free intercept of item MOTS4.

^g^ Free intercept of item P2.
